# A Rare Tumor of Nasal Cavity: Glomangiopericytoma

**DOI:** 10.1155/2014/282958

**Published:** 2014-07-20

**Authors:** Aysegul Verim, Cigdem Kalaycik Ertugay, Cigdem Tepe Karaca, Pembegul Gunes, Shahrouz Sheidaei, Cagatay Oysu

**Affiliations:** ^1^Department of Otorhinolaryngology/Head and Neck Surgery, Haydarpasa Numune Education and Research Hospital, 34668 Istanbul, Turkey; ^2^Department of Otorhinolaryngology/Head and Neck Surgery, Istanbul Hospital, Baskent University, Kulak Burun Boğaz Kliniği, Altunizade, Mahir İz Caddesi No. 43, 34662 Istanbul, Turkey; ^3^Department of Pathology, Haydarpasa Numune Education and Research Hospital, 34668 Istanbul, Turkey

## Abstract

Glomangiopericytoma is a rare vascular neoplasm characterized by a pattern of prominent perivascular growth. A 72-year-old woman was admitted to our clinic complaining of nasal obstruction, frequent epistaxis, and facial pain. A reddish tumor filling the left nasal cavity was observed on endoscopy and treated with endoscopic excision. Microscopically, closely packed cells interspersed with numerous thin-walled, branching staghorn vessels were seen. Glomangiopericytoma is categorized as a borderline low malignancy tumor by WHO classification. Long-term follow-up with systemic examination is necessary due to high risk of recurrence.

## 1. Introduction

Glomangiopericytoma (GPC) is a rare vascular neoplasm of the nasal cavity and paranasal sinuses characterized by a pattern of prominent perivascular growth [1]. GPC comprises less than 0.5% of all sinonasal neoplasia [2]. Synonyms include sinonasal-type hemangiopericytoma, hemangiopericytoma-like tumor, and hemangiopericytoma of sinonasal origin [3, 4].

GPC was first reported as hemangiopericytoma (HPC) in 1942 [5]. Since its initial description, the definition of this disease has been questioned. HPC of sinonasal origin is clinically and pathologically different from the soft tissue HPC. Thompson et al. analyzed 104 cases of HPC located in the sinonasal cavity and noted first sinonasal-type HPC description [3]. The World Health Organization (WHO) classified this tumor as GPC in 2005. The etiology of GPC remains unknown; however past trauma, hypertension, pregnancy, and use of corticosteroids are considered predisposing factors [6].

We report a case of GPC of the left nasal cavity filling olfactory fissure area in a 72-year-old female treated with surgery in Haydarpasa Numune Education and Research Hospital.

## 2. Case Report

**Figure 1:**
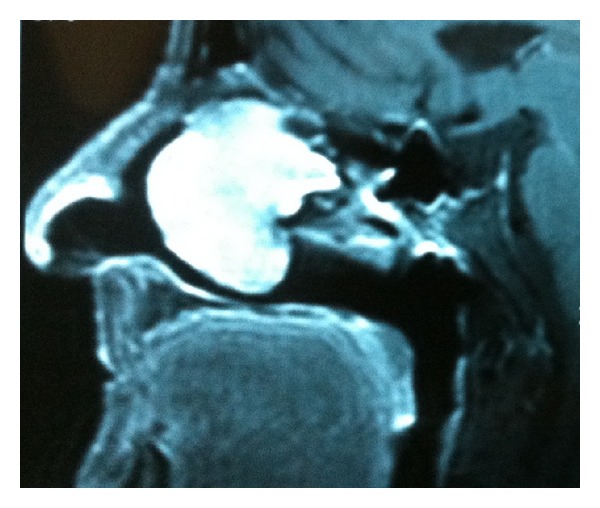
Sagittal MRI view of glomangiopericytoma extending along the olfactory fissure area.

**Figure 2:**
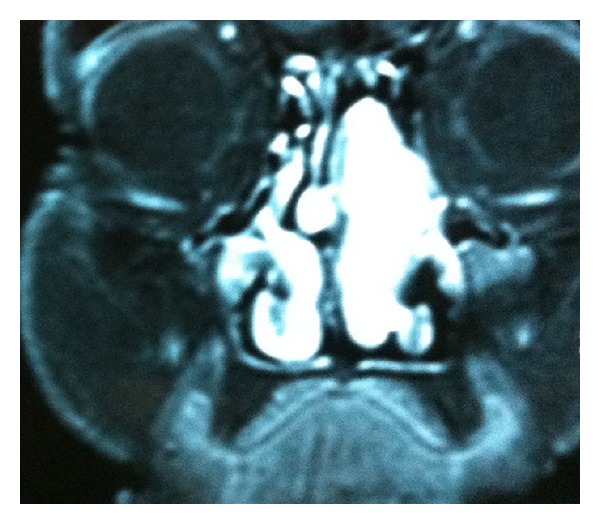
Coronal MRI view of glomangiopericytoma filling the left nasal cavity.

A 72-year-old woman attended our clinic complaining of nasal obstruction, frequent epistaxis, and facial pain for one year. She had no predisposing factor except hypertension. A reddish tumor filling the left nasal cavity was observed on endoscopy. MRI scan revealed a mass in left nasal cavity extending along the olfactory fissure area. Although the osteomeatal complex was involved, no particularity was observed on paranasal sinuses aeration. The tumor could not be distinguished from inferior and middle turbinates. The tumor was isointense (relative to normal nasal mucosa) on T1-weighted sequences and isointense-to-high signal intensity on T2-weighted sequences (Figures 1 and 2). A biopsy was taken and histologic examination of the specimen was reported as GPC. Regarding the location and moderate extension of the tumor the patient was scheduled for endoscopic surgery. A red colored, unilocular bleeding mass with a smooth surface was observed on endoscopy prior to surgery. This mass was invading the olfactory fissure area and was extending to the choana. However the osteomeatal complex was blocked, and paranasal sinuses were all free of disease.

Having in mind the abundant vascular supply of GPC bipolar cautery was used for devascularization and reduction of the tumor volume to prevent hemorrhage. After shrinkage of the mass with cauterization, resection was achieved with a microdebrider. No complication or CSF leak was observed at the end of the operation. The amount of bleeding was about 20 ml (measured in the suction pump) at the end of complete endoscopic removal. Intranasal tampooning with antibiotic ointment was applied for two days. No recurrence has been observed on her 2-year follow-up endoscopy.

**Figure 3:**
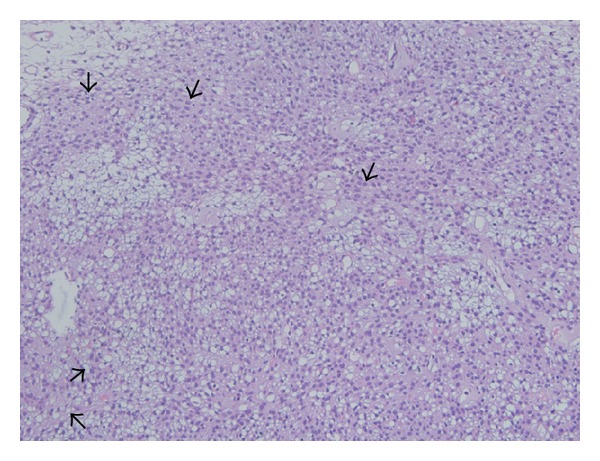
Spindle cell tumor proliferation embedded with different caliber vessels in submucosa (20 × 10 HE). Spindle cells are indicated with black arrow head.

**Figure 4:**
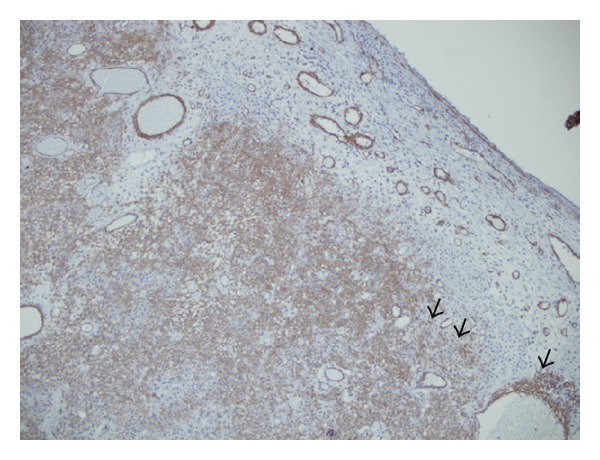
Tumor cells are strongly positive to *α*-smooth muscle actin (20 × 10 SMA). Spindle cells are indicated with black arrow head.

Hematoxylin and eosin staining of the specimen showed that the tumor was covered with normal respiratory epithelium. Tumor with solid and diffuse pattern was localized in submucosa. Different caliber vessels were embedded within the tumor (Figure 3). Some vessels had a staghorn appearance. The proliferated tumor cells were composed of each uniform and oval to spindle-shaped cell with a round nucleus, large eosinophilic cytoplasm and without nucleolus. An immunohistochemical study was performed using the Dako Envision method. The tumor cells were strongly positive to vimentin, *α*-smooth muscle actin (Figure 4), and muscle specific actin and negative to periodic acid shift, periodic acid shift diastase, PAN cytokeratin, low molecular weight cytokeratin, epithelial membrane antigen, S-100 protein, and CD 34.

## 3. Discussion

GPC is a rare mesenchymal tumor arising almost exclusively from the nasal cavity or paranasal sinuses and characterized by a pattern of prominent perivascular growth [1, 7]. GPC comprises less than 0.5% of all sinonasal neoplasia [4]. The peak incidence is during the sixth or seventh decade with a slight female predominance [8, 9]. The most common symptoms are epistaxis and/or nasal obstruction [3, 10]. Radiological examinations reveal opacification caused by polypoid mass, rarely with bone invasion [3]. Although past trauma, hypertension, pregnancy, and use of corticosteroids are considered predisposing factors, the etiology is not clear [6]. The treatment is complete surgical resection [8, 10, 11]. Because the tumor is highly vascular, some authors advocate preoperative embolization [12–14].

The patient of this report had one year history of nasal obstruction, frequent epistaxis, and facial pain. She had no predisposing factor except hypertension. Excision of tumor with endoscopic surgery was performed without preoperative embolization. Furthermore the most important point regarding this report is that the tumor was filling the olfactory fissure area and elongating to left nasal cavity.

Diagnosis was done by histologic examination. The tumor was comprised of closely packed cells and sometimes exhibiting storiform, whorled, or palisaded pattern, interspersed with numerous thin-walled, branching staghorn vessels. The neoplastic cells were uniform and possessed round to oval to spindle-shaped nuclei [4]. Immunohistochemical study showed that the tumor cells were strongly positive to vimentin, *α*-smooth muscle actin, and muscle specific actin and negative to CD34.

Differential diagnosis includes solitary fibrous tumor, myopericytoma, glomus tumor, and synovial sarcoma.

Solitary fibrous tumor is rich in collagen and, differently from GPC, tumor cells are positive to bcl-2 and negative to CD34 and actin.

The tumor cells of myopericytoma have a myxoid stroma with a solid growth pattern. It is differentiated from GPC by its morphologic character as concentric perivascular proliferation of spindle cells.

Glomus tumor is different from GPC morphologically as it does not have staghorn-like vessel proliferation and it is usually localized in distal extremities.

Synovial sarcoma is a biphasic tumor that it has both epithelial and mesenchymal components.

Regarding the histopathologic pattern of the resected tumor, the final diagnosis was certainly GPC. Since its definition as GPC in 2005, only a few cases of GPC have been described [7, 10, 13, 15].

Although it is categorized as a borderline low malignancy tumor by WHO classification, a minority can recur or can be fatal [3]. It is believed that recurrence might be a consequence of incomplete initial excision. In the present case, the tumor was completely resected so we consider that the patient is now completely cured and the potential for recurrence is low; however long-term follow-up with systemic examination is needed.
